# The effects of resistant starches on inflammatory bowel disease in preclinical and clinical settings: a systematic review and meta-analysis

**DOI:** 10.1186/s12876-020-01516-4

**Published:** 2020-11-10

**Authors:** Joshua Montroy, Rania Berjawi, Manoj M. Lalu, Eyal Podolsky, Cayden Peixoto, Levent Sahin, Alain Stintzi, David Mack, Dean A. Fergusson

**Affiliations:** 1 Clinical Epidemiology Program, Ottawa Hospital Research Institute, Ottawa, Canada; 2 School of Epidemiology and Public Health, University of Ottawa, Ottawa, Canada; 3 Department of Anesthesiology and Pain Medicine, The Ottawa Hospital, University of Ottawa, Ottawa, Canada; 4 Department of Biochemistry, Microbiology and Immunology, University of Ottawa, Ottawa, Canada; 5 Inflammatory Bowel Disease Centre, Children’s Hospital of Eastern Ontario, Ottawa, Canada; 6 Centre for Practice-Changing Research, Office L1298a, 501 Smyth Road, Box 201B, Ottawa, ON K1H 8L6 Canada

**Keywords:** Inflammatory bowel disease, Resistant starch, Systematic review

## Abstract

**Background:**

Inflammatory bowel disease (IBD) is a debilitating chronic disease with limited treatment options. Resistant starches may represent a novel treatment for IBD. However, its efficacy and safety remain unclear. Our objective was to perform a systematic review to summarize the preclinical and clinical effects of resistant starch, which may help guide future studies.

**Methods:**

Medline, EMBASE, and the Cochrane Central Register were searched. Included studies investigated the use of resistant starch therapy in in vivo animal models of IBD or human patients with IBD. Articles were screened, and data extracted, independently and in duplicate. The primary outcomes were clinical remission (clinical) and bowel mucosal damage (preclinical).

**Results:**

21 preclinical (n = 989 animals) and seven clinical (n = 164 patients) studies met eligibility. Preclinically, resistant starch was associated with a significant reduction in bowel mucosal damage compared to placebo (standardized mean difference − 1.83, 95% CI − 2.45 to − 1.20). Clinically, five studies reported data on clinical remission but clinical and methodological heterogeneity precluded pooling. In all five, a positive effect was seen in patients who consumed resistant starch supplemented diets. The majority of studies in both the preclinical and clinical settings were at a high or unclear risk of bias due to poor methodological reporting.

**Conclusions:**

Our review demonstrates that resistant starch is associated with reduced histology damage in animal studies, and improvements in clinical remission in IBD patients. These results need to be tempered by the risk of bias of included studies. Rigorously designed preclinical and clinical studies are warranted.

Trial registration

The review protocols were registered on PROSPERO (preclinical: CRD42019130896; clinical: CRD42019129513).

## Background

Inflammatory bowel disease (IBD) is a chronic inflammatory condition, the prevalence of which is increasing worldwide [1, 2]. The two dominant subtypes of IBD are ulcerative colitis (UC) and Crohn’s disease (CD), which can differ in the location of inflammation within the digestive tract and clinical symptoms but also can have colonic disease involvement and overlapping symptomatology. Current available treatment options include aminosalicylates [3], immunomodulators [4], corticosteroids [5], biologic agents [6], dietary changes [7], and surgical interventions [8]. However, none of these treatments are curative, and have been known to be associated with adverse effects [9–11]. Effective, less costly and more tolerable treatment options are currently needed in the long-term treatment of IBD.

Dietary fibres, such as resistant starches (RS) are a promising therapeutic for IBD [12]. Resistant starches avoid digestion in the small intestine and are subsequently fermented in the large intestine [13]. Since resistant starch is a natural source of fibre commonly found in many foods (i.e. potatoes, plantains and legumes), it is easily accessible and may provide an attractive treatment option (especially in regions of the gut where the microbial fermentation of RS occurs) due to high tolerability and few if any adverse effects compared to pharmaceutical options. They are classified into five categories (RS 1–5) based on their chemical and physical properties. In pre-clinical animal models of IBD, resistant starch has demonstrated the ability to improve the microbiome by increasing the concentration of short-chain fatty acids and decreasing gut pH level, which provides a less favourable environment for microbial pathogens and pathobionts to thrive [14]. They have also been shown to have a positive effect on inflammation in IBD (i.e. decreases in both inflammatory cell infiltration and circulating cytokine levels) [15–17]. In the clinical human trials, evidence has been mixed, with RS being found to reduce diarrhea, constipation and induce tissue repair in individuals with IBD [7], but demonstrating no positive effects in others [18, 19].

In healthy adults ingesting RS, systematic reviews evidence demonstrates an increased fecal wet weight, and butyrate concentration while decreasing fecal pH [20]. However, the evidence of potential efficacy and safety of RS in the treatment of IBD has yet to be formally synthesized in either the clinical human or preclinical (i.e. animal model) settings. Performing an evaluation of existing preclinical and clinical evidence of the effects of RS for IBD will provide a current overview of RS therapy across the preclinical to clinical translational spectrum, help identify knowledge gaps and guide the design of future investigations. Therefore, the purpose of this review is to evaluate the effect of RS on animals and humans with IBD by measuring clinical remission and histopathological changes compared to other treatments or placebo.

## Methods

The review protocols were registered on PROSPERO (preclinical: CRD42019130896; clinical: CRD42019129513). This manuscript followed the reporting guidelines set by Preferred Reporting Items for Systematic Reviews and Meta-Analyses (PRISMA) statement [21].

### Eligibility criteria

Eligibility criteria for preclinical animal studies included controlled comparison studies that investigated the effect of RS compared to placebo, alternative treatments, or no active treatment in in vivo animal models of IBD. Exclusion criteria included animal models that do not represent IBD, studies with no comparison group, and in vitro or ex vivo studies. For human clinical studies, we included all interventional studies of IBD patients administered any form of RS. Interventional studies did not need to include a comparator arm. Observational studies, case reports, and case series were excluded. Only full text studies were considered (i.e., unpublished grey literature, abstracts, conference abstracts, commentaries, letters, reviews and editorials were excluded).

### Outcomes

The primary outcome for the preclinical studies was mucosal damage as assessed by histology. The secondary outcomes were myeloperoxidase activity (a measure of neutrophil infiltration), short-chain fatty acid production, and body weight. Tertiary outcomes were circulating cytokine levels and gut microbiome changes. The primary outcome of interest for clinical studies was clinical remission or response rates. The secondary outcomes included stool consistency and frequency, short-chain fatty acid production and inflammation (C-reactive protein). Tertiary outcomes included adverse events, withdrawal due to adverse events, and serious adverse events.

### Search strategy

We conducted two systematic literature searches (i.e. preclinical and clinical) in collaboration with an information specialist (Risa Shorr, MLS, Learning Services, The Ottawa Hospital). Both searches were conducted on MEDLINE (OVID interface, including In-Process and Epub Ahead of Print) and Embase (OVID interface), and additional searches were done to identify clinical studies in the ClinicalTrials.gov and Cochrane Central Register of Controlled Trials (Wiley interface). A Peer Review of Electronic Search Strategies (PRESS) was performed by a second information specialist who was not associated with the project [22]. The clinical and preclinical searches were performed on August 27, 2020 and August 26, 2020, respectively. There were no restrictions on language or year of publication. We examined reference lists of included clinical studies and relevant reviews identified through the search, in order to identify any additional relevant references. The complete search strategies can be found in Additional file 1: Appendix I.

### Study selection and data extraction

Abstract/title, full text screening and data extraction was done in duplicate by two independent reviewers using pre-established eligibility criteria. Data extraction forms for both clinical and preclinical studies were pilot-tested on five studies prior to proceeding to extracting data from all studies to ensure agreement between reviewers. Disagreements between reviewers at any stage of the review were resolved by discussion or with a third-party member if a consensus could not be reached. Data items extracted included study population and intervention characteristics, along with data pertaining to our outcomes of interest, and risk of bias details.

### Risk of bias assessment

The Systematic Review Center for Laboratory Animal Experimentation (SYRCLE) risk of bias tool was used in the preclinical review and the Cochrane Risk of Bias (RoB 2.0) was used for the clinical review [23, 24]. Risk of bias was assessed in duplicate by two independent reviewers in each of the two reviews. Disagreements were resolved first by discussion and if consensus was not obtained, a consulting a third-party member made the final judgement. Graphical representations of risk of bias of included studies were conducted using RevMan 5.3 (Cochrane Collaboration, Oxford, United Kingdom).

### Data analysis

Studies were pooled using Comprehensive Meta-Analyst (version 3; Biostat Inc., USA). For continuous outcomes, a mean difference (MD) or standardized mean difference (SMD) was calculated, dependent on the outcome. MD and SMD were calculated using random effects inverse variance meta-analyses and presented with accompanying 95% confidence intervals. SMD was used to analyze outcomes where heterogeneity exists in the method of outcome measurement (i.e. differing scales). Dichotomous outcomes were analyzed using a random effects meta-analysis based on the DerSimonian Laird model, and reported as risk ratios with 95% confidence intervals (CI). Statistical heterogeneity was assessed using the Cochrane I^2 ^statistic. The thresholds for interpretation of I^2^ were as follows: 0–40% low heterogeneity, 30–60% moderate heterogeneity, 50–90% may represent substantial heterogeneity, and 75–100% is considerable heterogeneity. Data not suitable for inclusion in meta-analyses were presented descriptively. The presence of publication bias was assessed using funnel plots, where sufficient data were available. Where sufficient data were available, we performed a priori defined subgroup analyses for the preclinical studies, including type of RS, source of RS (food source vs. pure form), and animal species.

## Ethical considerations

Ethics approval was not needed for the conduct of this study.

## Results

**Fig. 1:**
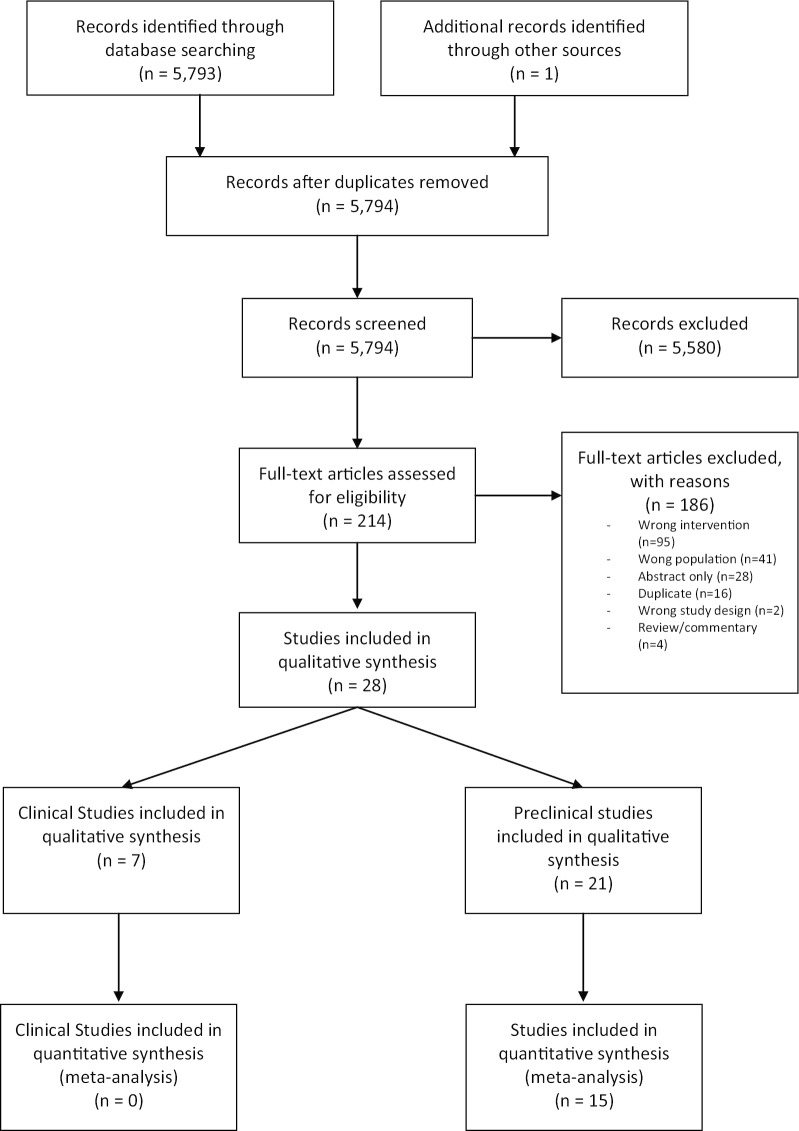
Study selection flow diagram

The literature searches yielded a total of 5,794 unique studies for title and abstract screening. Following independent, duplicate screening of abstract/titles then full-texts, 21 preclinical studies [15–17, 25–42] and seven clinical trials [43–49] met inclusion criteria (Fig. 1). The Clinicaltrials.gov search for the clinical systematic review yielded 201 trials, however none met the inclusion criteria of this review (see Additional file 1: Appendix II for details).

### Study characteristics

The 21 preclinical studies were published between 1999 and 2020 from 10 different countries (Table 1). All studies were performed in rodent models (rats, n = 10 and mice, n = 11). The age range for animals was 6 to 12 weeks, and the majority of studies were conducted using male animals only (n = 11). Thirteen articles reported studying acute colitis models rather than chronic models. IBD models used included; dextran sodium sulfate (DSS)-induced (n = 15), trinitrobenzene sulfonic acid (TNBS)-induced (n = 4), interleukin (IL)-10 knockout (n = 1), and CD4+ CD4RB T-cell transfer induced (n = 1).

**Table 1:** Characteristics of included preclinical studies

References	Host country(ies)	Sample size	Species	IBD model, chronicity	Age (weeks)	Biological sex (M/F)
Araki et al. [41]	Japan	40	Rats	DSS-induced, NR	12	M
Araki et al. [29]	Japan	18	Rats	DSS-induced, NR	11	M
Bassaganya-Riera et al. [34]	USA	275	Mice	IL-10 knockout, NR	8	50:50, M/F
Islam et al. [27]	Japan	24	Mice	DSS-induced, acute	10–12	M
Jacobasch et al. [36]	Germany	NR	Rats	TNBS-induced, NR	NR	NR
Kanauchi et al. [39, 40]	Japan	8	Rats	DSS-induced, acute	NR	M
Kanauchi et al. [39, 40]	Japan	8	Rats	DSS-induced, chronic	NR	M
Kanauchi et al. [38, 45]	Japan	20	Mice	DSS induced, acute	9	F
Kanauchi et al. [35]	Japan	16	Mice	CD4+ CD45RB T cell transfer, chronic	5	F
Komiyama et al. [32]	Japan	30	Mice	DSS-induced, acute	9	F
Le Leu et al. [15]	Australia	32	Mice	DSS-induced, acute	NR	M
Majumder et al. [28]	Canada & Japan	24	Mice	DSS-induced, acute	6–8	F
Moreau et al. [17]	France	72	Rats	DSS-induced, chronic	NR	M
Moreau et al. [37]	France	60	Rats	DSS-induced, acute and chronic	NR	M
Morita et al. [16]	Japan	32	Rats	TNBS-induced, acute	NR	M
Panasevich et al. [26]	USA	66	Mice	DSS-induced, acute	8–10	M
Praengam et al. [25]	Thailand	32	Mice	DSS-induced, acute	6	F
Qian et al. [30]	China	40	Mice	DSS-induced, acute	7	F
Rodriguez-Cabezas et al. [33]	Spain	50	Rats	TNBS-induced, acute	NR	F
Scarminio et al. [31]	Brazil	72	Rats	TNBS-induced, acute	NR	M
Shinde et al. [42]	Australia	50	Mice	DSS-induced, acute	7	Both

*DSS* dextran sulfate sodium, *F* female, *M* male, *NR* not reported, *SCID* severe combined immunodeficiency, *TNBS* trinitrobenzene sulfonic acid

The seven clinical studies were published between 1995 and 2015, with three studies from Japan, two from Australia, and one each from Sweden and the United Kingdom (Table 2). The total number of participants in all seven studies was 164 subjects (median 21, range 6–59). Two studies were single arm trials administering RS, two were single arm cross-over trials comparing different regimens of RS administration, two were two-arm trials comparing RS-supplemented diets to regular diets, and one study was a two-arm cross-over trial comparing high and low dose RS diets. All studies were in the adult population, with the majority of studies only including UC patients (n = 6), while one study had both UC and CD patients. Four studies recruited patients who were in a state of clinical remission, and one study recruited patients who had “no change in disease activity for at least 4-week prior to trial” regardless of actual disease status. Two studies recruited UC patients with no other specific inclusion criteria reported. Two studies reported the length of follow-up conducted (six months and two months).

**Table 2:** Characteristics of included clinical studies

References	# centres	Sample size (control/intervention)	Age of cohort (mean, range)	% Male (overall)	Follow-up	Disease status	Study design	Groups	
James et al. [49]	NR	29 (10/19)	41 (26–66)^†^ 38 (18–72)^‡^	41	NR	In remission	Two arm randomized cross-over trial	Ulcerative colitis patients	(i) High dose RS (ii) Low dose RS
								Healthy controls	(i) High dose RS (ii) Low dose RS
Clarke et al. [48]	NR	7	56 (37–81)	14	NR	In remission	Single arm, cross over	Ulcerative colitis & crohn’s disease patients	(i) HAMS (ii) LAMS (iii) Acetylated HAMS (iv) Propionylated HAMS (v) Butyrylated HAMS
Hanai et al. [46]	3	59 (37/22)	40.7 (2.3)^†§^ 42.5 (2.9)^‡§^	58	NR	In remission	Two-arm	Ulcerative colitis patients	GBF-supplemented diet
									Regular diet
Hallert [47]	3	32 (10/22)	43 (21–64)^†^ 44 (20–77)^‡^	59	6 months	In remission	Two-arm	Ulcerative colitis patients	Oat bran-supplemented diet
									Regular diet
Kanauchi [38, 45]	8	21	42.5 (2.9)^§^	NR	NR	No change in disease activity for at least 4-weeks prior to trial	Single arm	Ulcerative colitis patients	GBF-supplemented diet
Silvester et al. [43]	1	6	50 (39–59)	17	NR	Unclear	Randomized single arm, cross over trial	Ulcerative colitis patients	(i) High RS (ii) Regular diet (iii) Potato flour (iv) Low RS (v) Medium RS
Mitsuyama et al. [44]	1	10	44.1 (26–67)	50	2 months	Unclear	single arm	Ulcerative colitis patients	GBF-supplemented diet

*GBF* germinated barley foodstuff, *HAMS* high-amylose maize starch, *LAMS* low-amylose maize starch, *N/A* not applicable, *NR* not reported, *RS* resistant starch

^§^Age mean (SEM)

^†^Age of control group

^‡^Age of intervention group

### Intervention characteristics

In preclinical studies, type 3 RS was the most commonly used (n = 7), while six studies used type 1 RS, sex used type 2 RS, and two studies used type 5 RS (Table 3). All studies gave the animals free access to either the RS-supplemented diet or control diet, and access to experimental diets ranged from 8 to 42 days. Seventeen studies reported inducing colitis in animals after administering dietary intervention, and four induced colitis during access to dietary intervention. Eight studies reported housing animals individually, and the remaining studies did not report on animal housing.

**Table 3:** Preclinical intervention characteristics

References	Type of RS	Source of RS	RS Dose	Frequency/duration	Disease induction timing	Animal housing
Araki [41]	1	GBF	34 g/100 g diet	11 days, free access to diet	3 days after access to diet	NR
Araki [29]	1	GBF	34 g/100 g diet	8 days, free access to diet	Same time as diet administration	NR
Bassaganya-Riera [34]	3	Promitor RS-75	4 g/100 g diet	47 days, free access to diet	IL-10 knock-out mice developed colitis throughout the study	NR
Islam [27]	3	Rice bran	10 g/100 g of feed	16 days, free access to diet	4 days after access to diet	NR
Jacobasch et al. [36]	2	RS	15.38 g/100 g of feed	35 days, free access to diet	2 weeks after access to diet	NR
Kanauchi et al. [39, 40]	1	GBF	34 g/100 g diet	12 days, free access to diet	1 week after access to diet	Individually
Kanauchi et al. [39, 40]	1	GBF	34 g/100 g diet	42 days, free access to diet	Same time as diet administration	Individually
Kanauchi et al. [38, 45]	1	GBF	34 g/100 g diet	13 days, free access to diet	1 week after access to diet	Individually
Kanauchi et al. [35]	1	GBF	34 g/100 g diet	9 weeks, free access to diet	2 weeks after access to diet	NR
Komiyama et al. [32]	3	Rice bran	4 g/100 g diet	13 days, free access to diet	1 week after access to diet	Individually
Le Leu et al. [15]	2	HAMS	5 g/100 g diet	12 days, free access to diet	Same time as diet administration	NR
Majumder et al. [28]	5	Isomaltodextran	0.5, 1.0, 2.5, and 5.0% (w/v)	23 days via drinking water	15 days after access to diet	NR
Moreau et al. [17]	3	Novelose 330	11.5 g/100 g diet	14 days, free access to diet	1 week after access to diet	Individually
Moreau et al. [37]	3	Novelose 330	11.5 g/100 g diet	14 days, free access to diet	1 week after access to diet	Individually
Morita et al. [16]	2	HACS	30 g/100 g diet	18 days, free access to diet	10 days after access to diet	Individually
Panasevich et al. [26]	2	Potato fibre	0.2 g/100 g diet	22 days, free access to diet	2 weeks after access to diet	Individually
Praengam et al. [25]	3	Brown rice and retrograded brown rice	BR: 9 g/100 g diet RBR: NR	28 days, free access to diet	2 weeks after access to diet	NR
Qian et al. [30]	3	RS 3 extract	7 g	14 days, free access to diet	1 week after access to diet	NR
Rodriguez-Cabezas et al. [33]	5	Maltodextrin	2 g/rat/day	14 days via drinking water	2 weeks after access to diet	NR
Scarminio et al. [31]	2	Green dwarf banana flour	7 g/100 g diet	21 days, free access to diet	2 weeks after access to diet	NR
Shinde et al. [42]	2	Green banana flour	0.4 g/mouse/day	14 days, free access to diet	1 week after access to diet	NR

*GBF* germinated barley foodstuff, *HAS* high amylose maize starch, Novelose 330, retrograded Hylon 7, and high amylo-cornstarch

Type 1 RS was most commonly investigated in clinical studies (n = 4), while one study used type 2, one study used a mix of types 1 and 2 and one study used a mix of types 2 and 3 (Table 4). Sources of RS included germinated barley foodstuff, high-amylose maize starch, oat bran, potatoes and bananas. The duration of RS intervention varied from 5 days to 24 weeks. Administration of RS varied among studies. The lowest dose of RS given to participants was 0.6 g/day, while the highest was 34.8 g/day. Of the three cross-over studies, only one mentioned a wash-out period (14 days).

**Table 4:** Clinical intervention characteristics

References	Type of RS	Source of RS	Dose of RS	Frequency and duration of administration	Concomitant therapies	Wash-out period
James et al. [49]	RS1, RS2	HAMS added to bread, cereal and muffins	5 g (low dose) 15 g (high dose)	In diet for 17 days (3 day ramp-up period of 25% of total increase per day)	Aminosalicylates immunomodulators Corticosteroids None	14 days
Clarke et al. [48]	RS 2	HAMS added to milk-based chocolate custards	20 g	Daily for 6 days	None	None
Hanai et al. [46]	RS 1	GBF, unclear administration	6.4 g	In diet daily for 12 months	Aminosalicylates Corticosteroids	N/A
Hallert et al. [47]	RS 1	4 slices of oat bran–enriched bread and 37 mL of oat bran suspended in water, juice, or yogurt	0.6 g	In diet daily for 12 weeks	Aminosalicylates Corticosteroids Immunosuppressive agent	N/A
Kanauchi et al. [38, 45]	RS 1	GBF	6.4–10.2 g	In diet daily for 24 weeks	Aminosalicylates Corticosteroids	N/A
Silvester et al. [43]	RS 2, RS3	13 different foods with about three-quarters of the amount fed in the MRS and HRS test diets from potato flour biscuits and bananas	High: 34.8 g (32.9–36.0) Medium: 17.3 g (16.5–17.9) Low: 2.9 g (2.6–3.2) Potato flour: 11.8 g	Diet period was 5 days (control, low, medium, high, potato for 1 day each)	Naproxen	NR
Mitsuyama et al. [44]	RS 1	GBF, oral administration	10.2 g	Daily (split 3 times a day) for 4 weeks	Aminosalicylates Corticosteroids	N/A

*GBF* germinated barley foodstuff, *HAMS* high-amylose maize starch, *N/A* not applicable

Adherence to the dietary intervention was reported in two of the seven clinical studies through diary entries, returned foods, checklist and compliance records. One study had an adherence of 88–100% in both control and intervention groups and the second study reported a compliance of at least 80% of dietary fiber given. Adherence was not measured in any of the included preclinical studies.

### Preclinical outcomes

#### Primary outcome

**Fig. 2:**
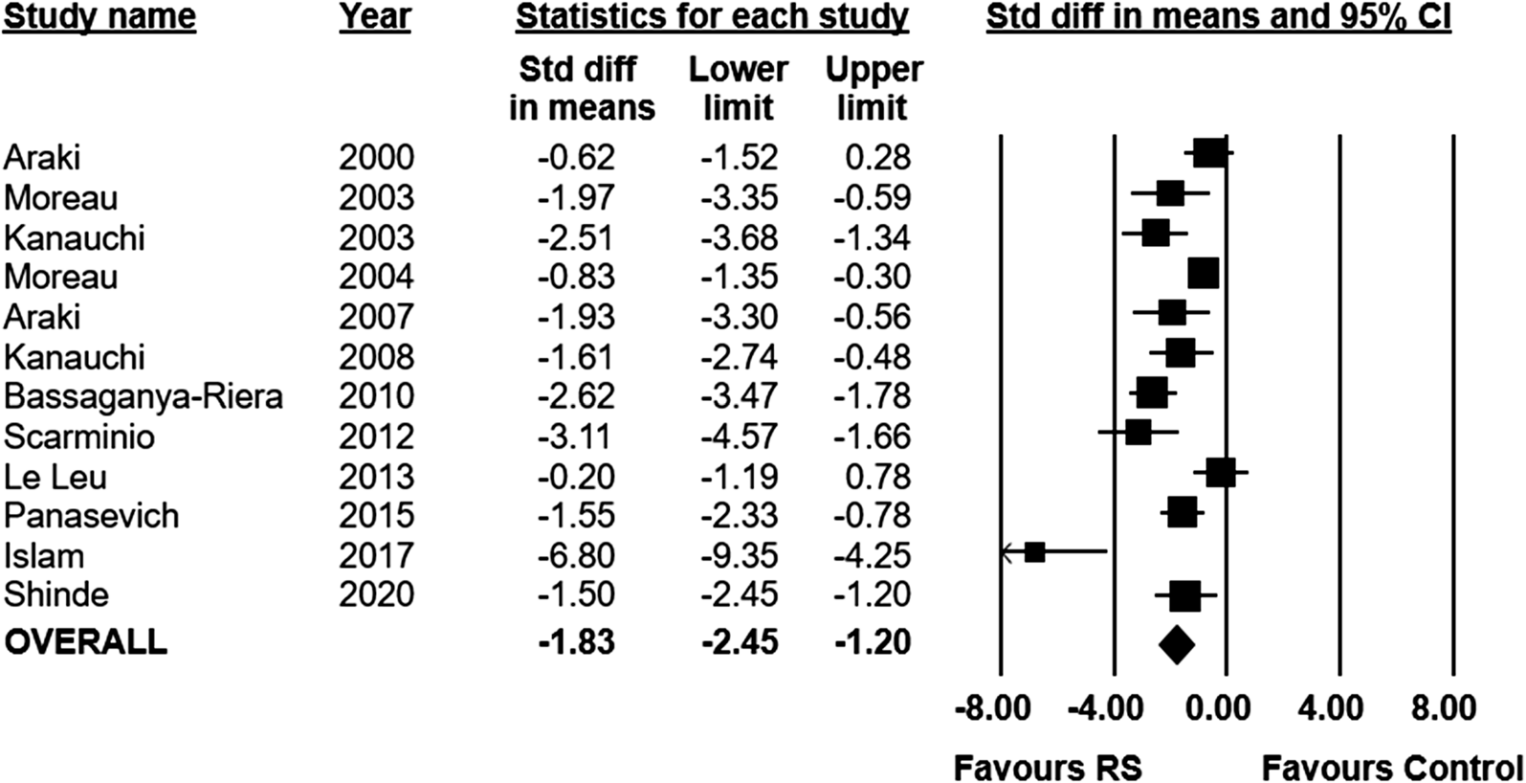
Standardized mean differences (95%) and pooled estimates for histology score (preclinical studies)

Eleven preclinical studies reported data on bowel mucosal damage as assessed by histology (n = 261 animals). Administration of RS was associated with a significant reduction in histological score compared to control (SMD − 1.83; 95% CI − 2.45 to − 1.20, I^2^ = 77%) (Fig. 2). Further details on the measurement of histology scores in individual studies can be found in the appendix (Additional file 1: Appendix III). A post-hoc sensitivity analysis was performed removing extreme values, given the presence of an outlier in the analysis. After the removal of extreme values, RS remained associated with improvements in histological score (Additional file 1: Appendix IV). A small degree of publication bias was indicated in the funnel plot and with eggers regression test, however this is largely driven by a single outlier (Additional file 1: Appendix V). In our a priori subgroup analyses, the effect of RS on histological scores did not vary by type of RS, source of RS (food vs pure), or animal species (Additional file 1: Appendix IV).

#### Secondary outcomes

**Fig. 3:**
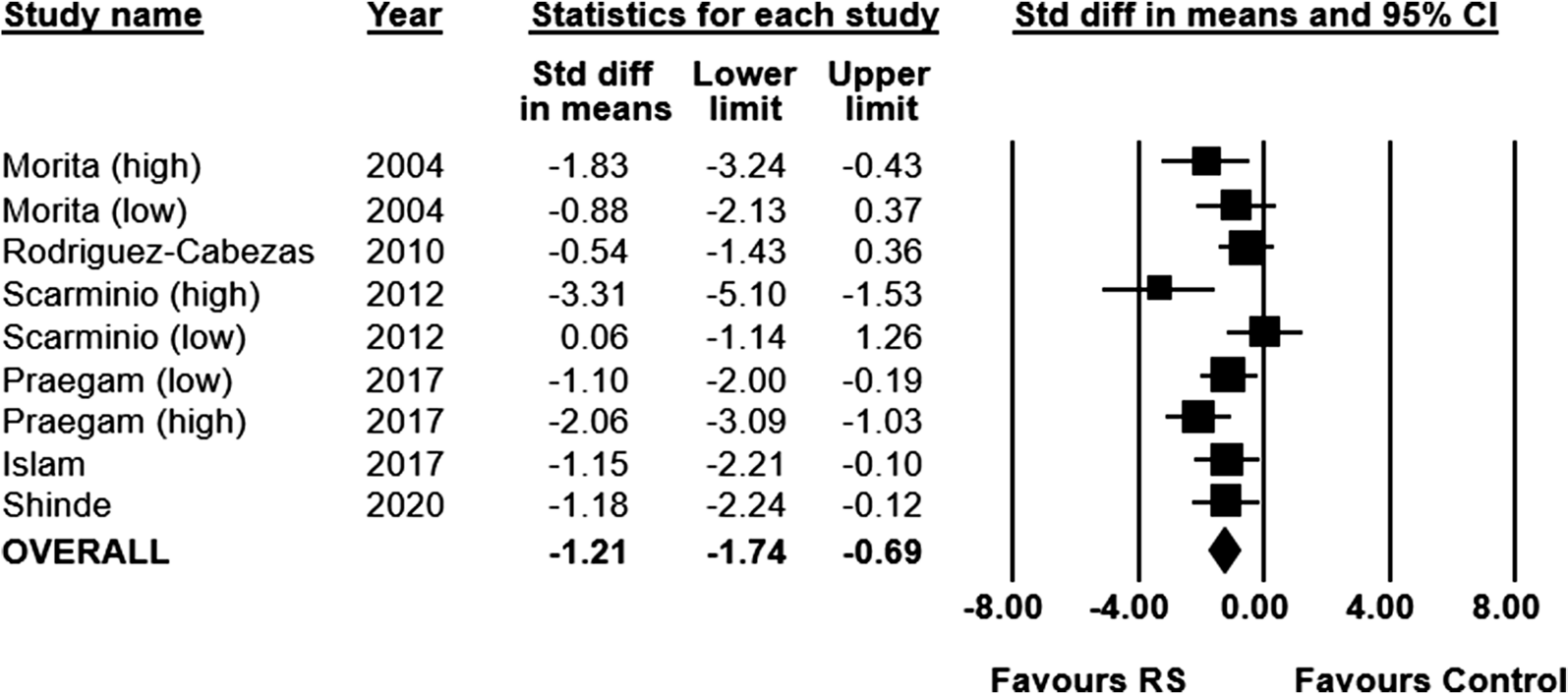
Standardized mean differences (95%) and pooled estimates for myeloperoxidase (preclinical studies)

MPO activity was measured in six of the included preclinical studies (n = 148 animals). Animals who received a RS supplemented diet had significantly decreased MPO concentration, compared to animals who received a control diet (SMD − 1.21; 95% CI − 1.74 to − 0.69, I^2^ = 48%) (Fig. 3).

**Fig. 4:**
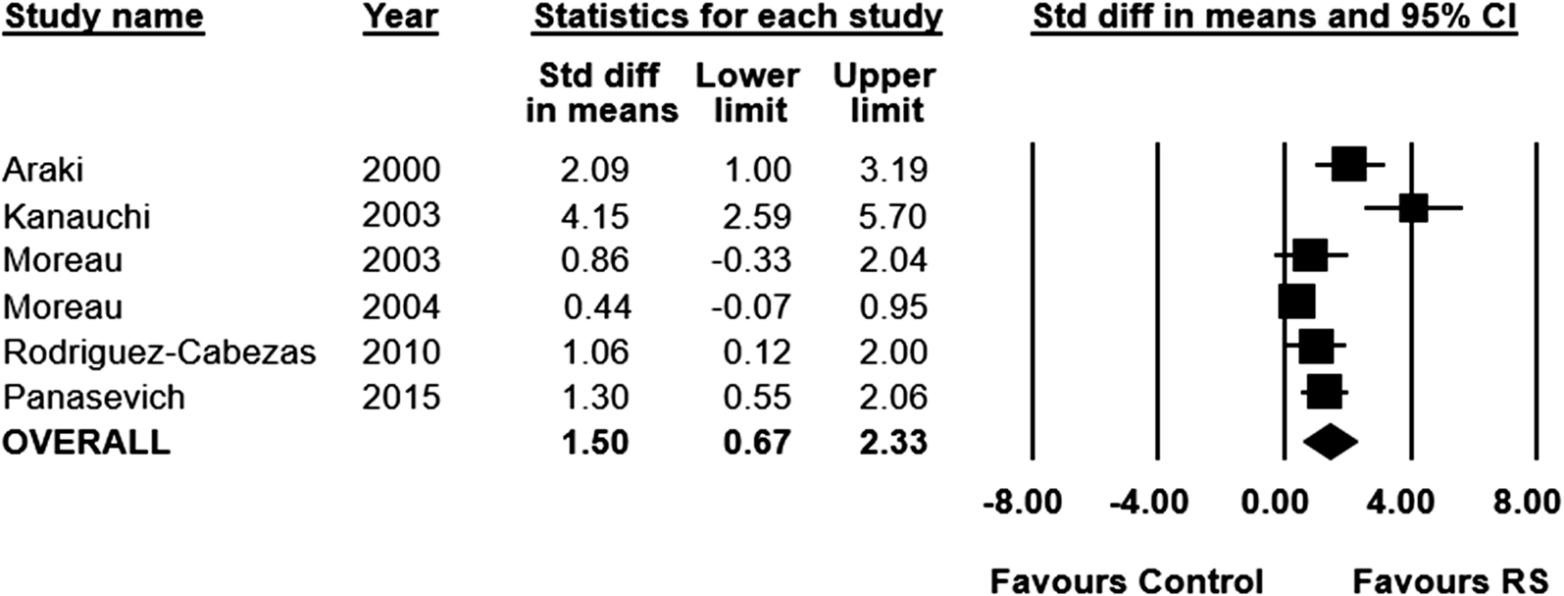
Standardized mean differences (95%) and pooled estimates for short chain fatty acid (preclinical studies)

Short-chain fatty acid concentration was measured in nine of the included studies (n = 223 animals). Three studies did not report data in a manner suitable for inclusion in the meta-analysis. Short chain fatty acid concentration increased significantly in RS fed animals, compared to control (SMD 1.50; 95% CI 0.67 to 2.33, I^2^ = 80%) (Fig. 4).

**Fig. 5:**
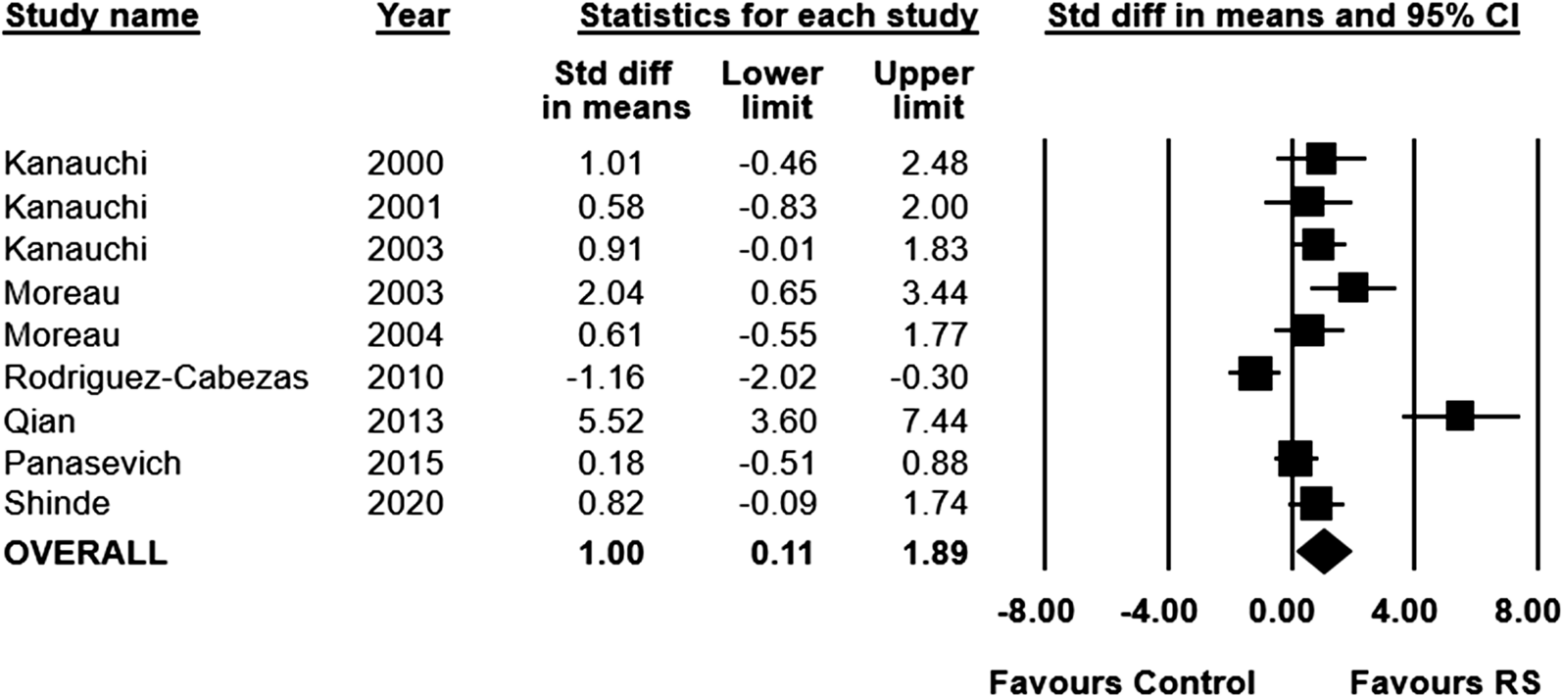
Standardized mean differences (95%) and pooled estimates for body weight (preclinical studies)

Body weight was measured in ten of the included studies (n = 253 animals), with one study not reporting data in a format suitable for inclusion in the meta-analysis. Animals fed a RS-supplemented diet had significantly higher body weights at the end of the experimental period, compared to animals fed a control diet (SMD 1.00; 95% CI 0.11 to 1.89, I^2^ = 83%) (Fig. 5).

#### Tertiary outcomes

Seven of the included studies evaluated cytokine activity. A pooled analysis could not be performed due to the heterogeneity in outcome measurement and reporting. Cytokines measured included IL-6, IL-10, IFNy, TNF-a, TGF-B, and IL-1B. In six of the seven studies, the cytokine levels in the intervention groups were significantly lower than that of the control groups at the conclusion of the study. The remaining study showed no statistically significant differences between the RS and control groups, however, when RS and fructooligosaccharides were mixed together, a significant decrease in TNFa and IL-1b production was seen.

Three studies reported data on gut microbial changes. One study highlighted the decrease in colonic counts of lactobacilli and bifidiobacteria following colitis induction compared to non-colitic groups [33]. The same study demonstrated that none of the intervention groups were able to counteract the decrease in lactobacilli counts, however, the RS diet was able to decrease enterobacteria counts compared to the untreated controls. One study suggested that a combination of red meat and RS reduced *C. coccoides, Enterococcus spp.* and *E. coli* in animals at the end of the intervention (although statistical analyses were not performed) [15]. Another study demonstrated that mice fed brown rice and retrograded brown rice (high RS content) had a much more diverse microbiota than the mice fed white rice-fed mice (low RS content) [25].

### Clinical outcomes

#### Primary outcome

Five of the included clinical studies reported on clinical remission and response rates (n = 151 patients). Due to the heterogeneity of study designs and outcome measurement, no formal pooling of data was performed, and clinical remission and response data is presented descriptively for each of the five studies. A number of measures were used across the five studies, including the Clinical Activity Index (n = 4), Seo Activity Index (n = 1), recurrence rates (n = 1), and steroid sparing (n = 1). In two studies that included UC patients with active disease, a significant decrease in Clinical Activity Index scores was observed (at two months [44] and 24 weeks [45] after initiation of treatment). Of the three studies which were performed in UC patients in remission, all three demonstrated that patients remained in remission at the conclusion of the study (48 days [49], 6 months [47], 12 months [46] after initiation of treatment). One of these studies observed a decrease from baseline in the Clinical Activity Index score, recurrence rate and steroid sparing at 12 months. Complete remission and response score are outline in Table 3.

#### Secondary outcomes

Due to clinical heterogeneity in the reporting of secondary outcomes, no formal statistical analysis was performed, and results are presented descriptively. Four clinical studies measured short-chain fatty acid concentration, three of which showed a significant increase by the end of the intervention period in resistant starch-fed patients [43, 47–49]. The one study which saw no difference in overall short-chain fatty acid concentration from baseline, did observe an increase in butyrate alone however [47].

Two studies reported measuring stool consistency and or frequency. In one study, high RS and wheat bran was associated with a significantly shorter whole gut transit time in healthy control patients, but no significant difference was seen in UC participants [49]. The other study saw a “significant correlation between the proportion of RS and the solids recovered in effluent” [43].

One study reported levels of C-reactive protein [44]. No statistically significant change was noted, however the authors suggested there was a trend towards decreasing levels.

#### Tertiary outcomes

Adverse events were reported in four of the included clinical studies. Two studies reported no adverse events [44, 46]. The third study demonstrated that the RS supplemented diet was associated with a reduction in adverse events, including complaints of diarrhea, abdominal pain, and gastroesophageal reflux by the end of the study (12 weeks), when compared to a regular diet control group [47]. The fourth study found that “pain/cramps” and “bloating/wind” increased from baseline in UC patients in the low RS group, whereas the same effect was not seen in the high RS group, or the healthy controls [49].

### Risk of bias

The risk of bias for the preclinical studies is summarized in the appendix (Additional file 1: Appendix VI). The quality of studies was found to be poor, as all included studies having an unclear risk of bias across the majority of assessed domains. Adding to this, no studies reported their randomization scheme, or method of allocation concealment. Finally, no studies adequately described the baseline characteristics of included animals or reported randomly housing animals. Eight studies reporting blinding of outcome assessment, while no studies reported blinding of personnel.

Risk of bias for the clinical studies is summarized in the appendix (Additional file 1: Appendix VI). Overall, the methodological quality of studies appears poor, with all studies being found to be at a high risk of bias in at least one domain. One study reported the method of patient randomization, while none reported their method of allocation concealment. One study reported blinding of participants and personnel, while one study reported blinding of outcome assessment. All studies were judged to be at a low risk of bias for attrition bias. One study was found to be at a low risk of bias for selective reporting while three studies were at a low risk of other biases (conflicts of interest, funding, etc.). A breakdown of risk of bias for each individual study can be found in the appendix (Additional file 1: Appendix VI).

### Deviations from protocol

For clinical studies, no meta-analysis or subgroup analyses were undertaken due to insufficient reporting and clinical and methodological heterogeneity. Tertiary outcomes of withdrawals due to adverse events and the occurrence of serious adverse events could not be reported in this review due to a lack of data from the included primary studies. Planned subgroup analyses of animal model of IBD, dose of administration, and inflammatory bowel disease type were not performed due to insufficient availability data.

## Discussion

Acute and chronic inflammation of the intestinal mucosa defines IBD and there are a number of treatments now available. These include dietary approaches such as exclusive enteral nutrition and exclusion diets that have been found to have benefit in induction therapy of mild and moderate CD although evidence for maintenance of remission is lacking [50, 51]. The benefit derived from these approaches is thought to be mediated through the intestinal microbiome, although elucidation of such mechanisms remain largely unknown. Furthermore, given the great variance of the intestinal microbes between different individuals in health and disease it remains to be determined whether similar foods will have consistent benefit.

However, overall, the results of our systematic review and meta-analysis demonstrated that RS is associated with reduced mucosal damage in preclinical in vivo animal models lending plausibility given the benefits of animal models to remove confounders associated with human disease. Most of the animal models were DSS induced, most were in male animals and many of the parameters studied are consistent with human disease. Human clinical data was limited but small studies demonstrate that RS maintain clinical remission in patients with IBD and reduces the severity of symptoms associated with patients that have active disease. Additionally, both preclinical and clinical studies found that RS was associated with an increase in short-chain fatty acid production. Limited clinical data suggested that RS therapy was tolerable in IBD patients. Nonetheless, our review has demonstrated a continuity of evidence from the preclinical lab to early phase clinical trials performed to date.

Results from included clinical studies are in accordance with similar results observed in a systematic review of RS in a health adult population [20]. In this previous review, supplementing the diet with RS was found to have a beneficial effect on large bowel function in healthy adults, by increasing fecal wet weight and butyrate concentration, while decreasing fecal pH. In our current review focused on patients with IBD, while data on fecal wet weight and fecal pH were not reported, we did find a rise in short-chain fatty acid concentrations (including butyrate). In addition, our systematic review found the reporting of adverse events/tolerability to be suboptimal, with only three of nine studies reporting data on adverse events. The under-reporting of harms in primary studies [52, 53] is an issue that is compounded in systematic reviews [54, 55], which can then present a misconception that a particular treatment is safe/tolerable (RS in this scenario), when the evidence is actually uncertain. Although RS is a naturally occurring product and is likely to be safe, further research into the safety/tolerability of RS in an IBD population should be conducted.

Reporting of key methodological details was lacking for the preclinical studies, with only eight studies (38%) reporting blinding of outcome assessment, and none reporting the randomization of animals to treatment and control groups. This is particularly problematic as the absence of key methodological details (i.e. randomization and blinding), has been shown to be associated with increased effect sizes [56, 57]. Thus, it is possible that we are overestimating the beneficial effect of RS in the preclinical setting. Similarly, clinically, our conclusions are again limited by potentially poor methodological quality. Only one study properly reported the randomization of participants. While our review included single arm studies (which makes randomization irrelevant), only one study blinded the outcome assessor, which remains possible even in single arm studies. As with preclinical studies, the lack of proper randomization [58, 59] and blinding [60, 61] is consistently associated with larger treatment effects, especially when the outcomes are subjective (as are many in IBD) [60, 61].

Statistical and methodological heterogeneity was frequently observed in the included studies, and represent a major limitation when drawing conclusions from our data. In the preclinical studies, statistical heterogeneity was high (77%) in the analysis of our primary outcome. This heterogeneity was not explained by any of our subgroup analyses (i.e. RS type/source and species). This has the potential to be explained by individual differences in the gut microbiome, which exist between animals, despite of a tightly controlled laboratory environment. Clinically, significant variability in treatment effect was also observed. In agreement with this observation, a few recent studies have also noted that individuals varied in their response to RS [62–65]. These varied outcomes are likely due to each RS having a different interaction with each individual’s microbiota composition and/or functionality. Hence, a personalized dietary intervention by matching the type of RS to the host microbiota might be required for beneficial effects on the host.

Clinically, we also observed significant methodological heterogeneity with regards to study design. For example, included in our review were four single arm studies (two of which were cross-over studies involving multiple diets) and three two-arm studies (one of which was a cross-over study involving four groups, i.e. two different diets in both IBD patients and healthy controls). Due to significant methodological heterogeneity between studies, we could not perform a meta-analysis, which limits our conclusions regarding efficacy of the treatment. It does, however, demonstrate the paucity of data and the need for future high-quality randomized controlled studies.

## Conclusion

In conclusion, RS reduces histology scores within in vivo models of IBD in preclinical studies. RS was observed to improve clinical remission, increase short-chain fatty production, and was not associated with any adverse events, however, this conclusion is based off of few studies that were of low quality. More research with increased quality of methods reporting are needed to evaluate the impact of RS on improving the underlying pathophysiology of IBD. Studies should evaluate the impact of RS alongside standard therapy as a combination treatment to amplify the effect of treatment and consideration of the phase of treatment may also be of importance in providing insight into the role of RS in therapy of CD and UC.

## Supplementary information

**Additional file 1**. Supplementary Information.

## Data Availability

All data generated or analysed during this study are included in this published article [and its supplementary information files].

## Abbreviations

CD: Crohn’s disease
DSS: Dextran sulfate sodium
IBD: Inflammatory bowel disease
IL: Interleukin
MD: Mean difference
PRESS: Peer review of electronic search strategy
PRISMA: Preferred reporting items for systematic reviews and meta-analyses
RS: Resistant starch
SMD: Standardized mean difference
SYRCLE: Systematic Review Centre for Laboratory Animal Experimentation
TNBS: Trinitrobenzenesulfonic acid
UC: Ulcerative colitis
