# Supplementary material for: The effects of resistant starches on inflammatory bowel disease in preclinical and clinical settings: a systematic review and meta-analysis
Source: BMC Gastroenterol. 2020 Nov 10;20:372. doi: 10.1186/s12876-020-01516-4 (PMC7653724; doi:10.1186/s12876-020-01516-4)
Supplement: Supplementary file 1 — Additional file 1. Supplementary Information. [file 12876_2020_1516_MOESM1_ESM.docx]

**Appendix I – Search Strategy**

**Search Strategy for Clinical Systematic Review**

Database: Embase Classic+Embase <1947 to 2020 August 27>, Ovid MEDLINE(R) ALL <1946 to August 27, 2020>, EBM Reviews - Cochrane Central Register of Controlled Trials <July 2020>

Search Strategy:

--------------------------------------------------------------------------------

1     exp Starch/ (70435)

2     (Resistan* starch* or resistan* carbohydrate* or amylose or amylopectin or rs1 or rs2 or rs3 or rs4 or rs5).tw,kw. (14536)

3     (resistan* adj3 (starch* or carbohydrate*)).tw. (4135)

4     exp dietary fiber/ (39200)

5     (fiber or fibre or bran or roughage or prebiotic*).tw,kf. (399521)

6     or/1-5 (483449)

7     exp Digestive System Diseases/ (4759277)

8     (ibd or inflammat* bowel or irritable bowel or colitis or crohn* or intestin* inflammat* or gut inflammat*).tw,kw. (324989)

9     ((bowel or intestin* or colon*) adj (function or functions or health)).tw. (17179)

10     Feces/ (154760)

11     (fece* or fecal or faecal).tw,kw. (219584)

12     Defecation/ (19821)

13     exp colon/ (150402)

14     or/7-13 (5072383)

15     6 and 14 (36052)

16     randomized controlled trial.pt. (928745)

17     controlled clinical trial.pt. (183220)

18     random*.tw. (3059583)

19     placebo.ab. (669302)

20     clinical trials as topic.sh. (218237)

21     trial.ti. (681365)

22     clinical trial*.pt,tw,kw. (1793063)

23     intervention stud*.tw,kw. (50683)

24     or/16-23 (4577103)

25     15 and 24 (7108)

26     exp animals/ not humans/ (16933631)

27     25 not 26 (4914)

28     27 use medall (1994)

29     exp starch/ (70435)

30     (amylose or amylopectin or rs1 or rs2 or rs3 or rs4 or rs5).tw,kw. (12106)

31     (resistan* adj3 (starch* or carbohydrate*)).tw. (4135)

32     dietary fiber/ (37206)

33     (fiber or fibre or bran or roughage or prebiotic*).tw. (397971)

34     or/29-33 (481660)

35     digestive system disease/ or exp *digestive system disease/ or exp ulcerative colitis/ (3350580)

36     (ibd or inflammat* bowel or irritable bowel or colitis or crohn* or intestin* inflammat* or gut inflammat*).tw. (320792)

37     ((bowel or intestin* or colon*) adj (function or functions or health)).tw. (17179)

38     feces.mp. (254594)

39     (fece* or fecal or faecal).tw. (215544)

40     Defecation.mp. (35722)

41     colon/ (93944)

42     exp *intestine function/ (33011)

43     or/35-42 (3764325)

44     34 and 43 (30400)

45     random*.tw. or placebo*.mp. or double-blind*.tw. (3510196)

46     clinical trial/ (1483864)

47     intervention study/ (550596)

48     (clinical trial* or intervention stud*).tw. (930559)

49     trial*.ti. (845461)

50     or/45-49 (4983128)

51     44 and 50 (6738)

52     (exp animal/ or nonhuman/) not exp human/ (11206473)

53     51 not 52 (5778)

54     conference abstract.pt. (3225254)

55     53 and 54 (555)

56     53 not 55 (5223)

57     56 use emczd (2424)

58     exp Starch/ (70435)

59     (Resistan* starch* or resistan* carbohydrate* or amylose or amylopectin or rs1 or rs2 or rs3 or rs4 or rs5).tw,kw. (14536)

60     (resistan* adj3 (starch* or carbohydrate*)).tw. (4135)

61     exp dietary fiber/ (39200)

62     (fiber or fibre or bran or roughage or prebiotic*).tw,kf. (399521)

63     or/58-62 (483449)

64     exp Digestive System Diseases/ (4759277)

65     (ibd or inflammat* bowel or irritable bowel or colitis or crohn* or intestin* inflammat* or gut inflammat*).tw,kw. (324989)

66     ((bowel or intestin* or colon*) adj (function* or health)).tw. (17784)

67     Feces/ (154760)

68     (fece* or fecal or faecal).tw,kw. (219584)

69     Defecation/ (19821)

70     exp colon/ (150402)

71     or/64-70 (5072638)

72     63 and 71 (36067)

73     (conference or journal conference abstract or conference abstract).pt. (4063906)

74     72 and 73 (4201)

75     72 not 74 (31866)

76     74 use cctr (125)

77     75 use cctr (1408)

**78     28 or 57 or 77 (5826)  Medline, Embase, Cochrane**

**79     remove duplicates from 78 (3433)  Medline, Embase, Cochrane**

80     55 or 76 (642)

81     remove duplicates from 80 (544)

**82     79 use medall (1980)  Medline**

**83     79 use emczd (1164)  Embase**

**84     79 use cctr (289)  Cochrane**

**85     81 use emczd (509)  Embase Conferences**

**86     81 use cctr (35)           Cochrane Conferences**

**Search Strategy for preclinical Systematic Review**

Database: Embase Classic+Embase <1947 to 2020 August 26>, Ovid MEDLINE(R) ALL <1946 to August 26, 2020>
Search Strategy:
--------------------------------------------------------------------------------
1     exp Starch/ (70520)
2     (Resistan* starch* or resistan* carbohydrate* or amylose or amylopectin or rs1 or rs2 or rs3 or rs4 or rs5).tw,kw. (14625)
3     (resistan* adj3 (starch* or carbohydrate*)).tw. (3945)
4     exp dietary fiber/ (38183)
5     (fiber or fibre or bran or roughage or prebiotic*).tw,kf. (401058)
6     or/1-5 (484994)
7     exp Inflammatory Bowel Diseases/ (216448)
8     (ibd or inflammat* bowel or irritable bowel or colitis or crohn* or intestin* inflammat* or gut inflammat* or colon* inflammat*).tw,kw. (324467)
9     ((intestin* or gut or colon*) adj2 inflammat*).tw. (32439)
10     or/7-9 (362105)
11     6 and 10 (4711)
12     exp "animal experimentation"/ or exp "models, animal"/ or exp "invertebrates"/ or "Animals"/ or exp "animal population groups"/ or "chordata"/ or exp "chordata, nonvertebrate"/ or "vertebrates"/ or exp "amphibians"/ or exp "birds"/ or exp "fishes"/ or exp "reptiles"/ or "mammals"/ or "primates"/ or exp "artiodactyla"/ or exp "carnivora"/ or exp "cetacea"/ or exp "chiroptera"/ or exp "elephants"/ or exp "hyraxes"/ or exp "insectivora"/ or exp "lagomorpha"/ or exp "marsupialia"/ or exp "monotremata"/ or exp "perissodactyla"/ or exp "rodentia"/ or exp "scandentia"/ or exp "sirenia"/ or exp "xenarthra"/ or "haplorhini"/ or exp "strepsirhini"/ or exp "platyrrhini"/ or exp "tarsii"/ or "catarrhini"/ or exp "cercopithecidae"/ or exp "hylobatidae"/ or "hominidae"/ or exp "gorilla gorilla"/ or exp "pan paniscus"/ or exp "pan troglodytes"/ or exp "pongo pygmaeus"/ (32589897)
13     (animals or animal or mice or mus or mouse or murine or woodmouse or rats or rat or murinae or muridae or cottonrat or cottonrats or hamster or hamsters or cricetinae or rodentia or rodent or rodents or pigs or pig or swine or swines or piglets or piglet or boar or boars or "sus scrofa" or ferrets or ferret or polecat or polecats or "mustela putorius" or "guinea pigs" or "guinea pig" or cavia or callithrix or marmoset or marmosets or cebuella or hapale or octodon or chinchilla or chinchillas or gerbillinae or gerbil or gerbils or jird or jirds or merione or meriones or rabbits or rabbit or hares or hare or diptera or flies or fly or dipteral or drosphila or drosophilidae or cats or cat or carus or felis or nematoda or nematode or nematoda or nematode or nematodes or sipunculida or dogs or dog or canine or canines or canis or sheep or sheeps or mouflon or mouflons or ovis or goats or goat or capra or capras or rupicapra or chamois or haplorhini or monkey or monkeys or anthropoidea or anthropoids or saguinus or tamarin or tamarins or leontopithecus or hominidae or ape or apes or pan or paniscus or "pan paniscus" or bonobo or bonobos or troglodytes or "pan troglodytes" or gibbon or gibbons or siamang or siamangs or nomascus or symphalangus or chimpanzee or chimpanzees or prosimians or "bush baby" or prosimian or bush babies or galagos or galago or pongidae or gorilla or gorillas or pongo or pygmaeus or "pongo pygmaeus" or orangutans or pygmaeus or lemur or lemurs or lemuridae or horse or horses or pongo or equus or cow or calf or bull or chicken or chickens or gallus or quail or bird or birds or quails or poultry or poultries or fowl or fowls or reptile or reptilia or reptiles or snakes or snake or lizard or lizards or alligator or alligators or crocodile or crocodiles or turtle or turtles or amphibian or amphibians or amphibia or frog or frogs or bombina or salientia or toad or toads or "epidalea calamita" or salamander or salamanders or eel or eels or fish or fishes or pisces or catfish or catfishes or siluriformes or arius or heteropneustes or sheatfish or perch or perches or percidae or perca or trout or trouts or char or chars or salvelinus or "fathead minnow" or minnow or cyprinidae or carps or carp or zebrafish or zebrafishes or goldfish or goldfishes or guppy or guppies or chub or chubs or tinca or barbels or barbus or pimephales or promelas or "poecilia reticulata" or mullet or mullets or seahorse or seahorses or mugil curema or atlantic cod or shark or sharks or catshark or anguilla or salmonid or salmonids or whitefish or whitefishes or salmon or salmons or sole or solea or "sea lamprey" or lamprey or lampreys or pumpkinseed or sunfish or sunfishes or tilapia or tilapias or turbot or turbots or flatfish or flatfishes or sciuridae or squirrel or squirrels or chipmunk or chipmunks or suslik or susliks or vole or voles or lemming or lemmings or muskrat or muskrats or lemmus or otter or otters or marten or martens or martes or weasel or badger or badgers or ermine or mink or minks or sable or sables or gulo or gulos or wolverine or wolverines or minks or mustela or llama or llamas or alpaca or alpacas or camelid or camelids or guanaco or guanacos or chiroptera or chiropteras or bat or bats or fox or foxes or iguana or iguanas or xenopus laevis or parakeet or parakeets or parrot or parrots or donkey or donkeys or mule or mules or zebra or zebras or shrew or shrews or bison or bisons or buffalo or buffaloes or deer or deers or bear or bears or panda or pandas or "wild hog" or "wild boar" or fitchew or fitch or beaver or beavers or jerboa or jerboas or capybara or capybaras).tw. (10382260)
14     preclinic*.mp. (259106)
15     (preclinic* or pre clinic*).tw,kw. (248515)
16     or/12-15 (33908335)
17     11 and 16 (3360)
18     17 use medall (620)  Medline
19     exp starch/ (70520)
20     (amylose or amylopectin or rs1 or rs2 or rs3 or rs4 or rs5).tw,kw. (12296)
21     (resistan* adj3 (starch* or carbohydrate*)).tw. (3945)
22     dietary fiber/ or prebiotic agent/ (42148)
23     (fiber or fibre or bran or roughage or prebiotic*).tw. (399392)
24     or/19-23 (484877)
25     exp inflammatory bowel disease/ (216448)
26     (ibd or inflammat* bowel or irritable bowel or colitis or crohn*).tw. (311497)
27     ((colon* or gut or intestin*) adj3 inflammat*).tw. (41417)
28     or/25-27 (363237)
29     24 and 28 (5101)
30     exp animal experiment/ or exp animal model/ or exp experimental animal/ or exp transgenic animal/ or exp male animal/ or exp female animal/ or exp juvenile animal/ or animal/ or chordata/ or vertebrate/ or tetrapod/ or exp fish/ or amniote/ or exp amphibia/ or mammal/ or exp reptile/ or exp sauropsid/ or therian/ or exp monotremate/ or placental mammals/ or exp marsupial/ or Euarchontoglires/ or exp Afrotheria/ or exp Boreoeutheria/ or exp Laurasiatheria/ or exp Xenarthra/ or primate/ or exp Dermoptera/ or exp Glires/ or exp Scandentia/ or Haplorhini/ or exp prosimian/ or simian/ or exp tarsiiform/ or Catarrhini/ or exp Platyrrhini/ or ape/ or exp Cercopithecidae/ or hominid/ or exp hylobatidae/ or exp chimpanzee/ or exp gorilla/ or exp orang utan/ or (animal or animals or pisces or fish or fishes or catfish or catfishes or sheatfish or silurus or arius or heteropneustes or clarias or gariepinus or fathead minnow or fathead minnows or pimephales or promelas or cichlidae or trout or trouts or char or chars or salvelinus or salmo or oncorhynchus or guppy or guppies or millionfish or poecilia or goldfish or goldfishes or carassius or auratus or mullet or mullets or mugil or curema or shark or sharks or cod or cods or gadus or morhua or carp or carps or cyprinus or carpio or killifish or eel or eels or anguilla or zander or sander or lucioperca or stizostedion or turbot or turbots or psetta or flatfish or flatfishes or plaice or pleuronectes or platessa or tilapia or tilapias or oreochromis or sarotherodon or common sole or dover sole or solea or zebrafish or zebrafishes or danio or rerio or seabass or dicentrarchus or labrax or morone or lamprey or lampreys or petromyzon or pumpkinseed or pumpkinseeds or lepomis or gibbosus or herring or clupea or harengus or amphibia or amphibian or amphibians or anura or salientia or frog or frogs or rana or toad or toads or bufo or xenopus or laevis or bombina or epidalea or calamita or salamander or salamanders or newt or newts or triturus or reptilia or reptile or reptiles or bearded dragon or pogona or vitticeps or iguana or iguanas or lizard or lizards or anguis fragilis or turtle or turtles or snakes or snake or aves or bird or birds or quail or quails or coturnix or bobwhite or colinus or virginianus or poultry or poultries or fowl or fowls or chicken or chickens or gallus or zebra finch or taeniopygia or guttata or canary or canaries or serinus or canaria or parakeet or parakeets or grasskeet or parrot or parrots or psittacine or psittacines or shelduck or tadorna or goose or geese or branta or leucopsis or woodlark or lullula or flycatcher or ficedula or hypoleuca or dove or doves or geopelia or cuneata or duck or ducks or greylag or graylag or anser or harrier or circus pygargus or red knot or great knot or calidris or canutus or godwit or limosa or lapponica or meleagris or gallopavo or jackdaw or corvus or monedula or ruff or philomachus or pugnax or lapwing or peewit or plover or vanellus or swan or cygnus or columbianus or bewickii or gull or chroicocephalus or ridibundus or albifrons or great tit or parus or aythya or fuligula or streptopelia or risoria or spoonbill or platalea or leucorodia or blackbird or turdus or merula or blue tit or cyanistes or pigeon or pigeons or columba or pintail or anas or starling or sturnus or owl or athene noctua or pochard or ferina or cockatiel or nymphicus or hollandicus or skylark or alauda or tern or sterna or teal or crecca or oystercatcher or haematopus or ostralegus or shrew or shrews or sorex or araneus or crocidura or russula or european mole or talpa or chiroptera or bat or bats or eptesicus or serotinus or myotis or dasycneme or daubentonii or pipistrelle or pipistrellus or cat or cats or felis or catus or feline or dog or dogs or canis or canine or canines or otter or otters or lutra or badger or badgers or meles or fitchew or fitch or foumart or foulmart or ferrets or ferret or polecat or polecats or mustela or putorius or weasel or weasels or fox or foxes or vulpes or common seal or phoca or vitulina or grey seal or halichoerus or horse or horses or equus or equine or equidae or donkey or donkeys or mule or mules or pig or pigs or swine or swines or hog or hogs or boar or boars or porcine or piglet or piglets or sus or scrofa or llama or llamas or lama or glama or deer or deers or cervus or elaphus or cow or cows or bos taurus or bos indicus or bovine or bull or bulls or cattle or bison or bisons or sheep or sheeps or ovis aries or ovine or lamb or lambs or mouflon or mouflons or goat or goats or capra or caprine or chamois or rupicapra or leporidae or lagomorpha or lagomorph or rabbit or rabbits or oryctolagus or cuniculus or laprine or hares or lepus or rodentia or rodent or rodents or murinae or mouse or mice or mus or musculus or murine or woodmouse or apodemus or rat or rats or rattus or norvegicus or guinea pig or guinea pigs or cavia or porcellus or hamster or hamsters or mesocricetus or cricetulus or cricetus or gerbil or gerbils or jird or jirds or meriones or unguiculatus or jerboa or jerboas or jaculus or chinchilla or chinchillas or beaver or beavers or castor fiber or castor canadensis or sciuridae or squirrel or squirrels or sciurus or chipmunk or chipmunks or marmot or marmots or marmota or suslik or susliks or spermophilus or cynomys or cottonrat or cottonrats or sigmodon or vole or voles or microtus or myodes or glareolus or primate or primates or prosimian or prosimians or lemur or lemurs or lemuridae or loris or bush baby or bush babies or bushbaby or bushbabies or galago or galagos or anthropoidea or anthropoids or simian or simians or monkey or monkeys or marmoset or marmosets or callithrix or cebuella or tamarin or tamarins or saguinus or leontopithecus or squirrel monkey or squirrel monkeys or saimiri or night monkey or night monkeys or owl monkey or owl monkeys or douroucoulis or aotus or spider monkey or spider monkeys or ateles or baboon or baboons or papio or rhesus monkey or macaque or macaca or mulatta or cynomolgus or fascicularis or green monkey or green monkeys or chlorocebus or vervet or vervets or pygerythrus or hominoidea or ape or apes or hylobatidae or gibbon or gibbons or siamang or siamangs or nomascus or symphalangus or hominidae or orangutan or orangutans or pongo or chimpanzee or chimpanzees or pan troglodytes or bonobo or bonobos or pan paniscus or gorilla or gorillas or troglodytes).ti,ab. (14708436)
31     (preclinic* or pre clinic*).tw,kw. (248515)
32     30 or 31 (14813805)
33     29 and 32 (1554)
**34     33 use emczd (917)  Embase
35     18 or 34 (1537)
36     remove duplicates from 35 (1105)
37     36 use medall (618)  Medline
38     36 use emczd (487)  Embase**

**Appendix II – Clincaltrials.gov search results**

Records excluded, with reasons
(n = 201)

- Wrong population (n=141)
- Wrong intervention (n=60)

Records screened
(n = 201)

Trials included
(n = 0)

Records identified
(n = 201)

**Appendix III. Additional histological data**

| First Author (year) | Type of Histological Assessment | Assessor(s) | Section(s) of bowel | Histology Score/ description (control) | Histology Score/ description  (intervention) | Outcome ascertainment window |
| --- | --- | --- | --- | --- | --- | --- |
| Araki (2000) | Histology via damage score | Two blinded assessors | Distal and middle portion of the colon | Distal: 8.75**±**1.10  Middle: 6.03**±**0.879 | Distal: 6.79**±**0.88 Middle: 2.58**±**0.152 | 8 days after disease induction, 11 days after intervention start |
| Araki (2007) | Mucosal damage score | Two independent and blinded investigators | Middle | 5.75 ± 0.844 | 2.80 ± 0.262 | 8 days after fed diet and disease induction |
| Bassaganya-Riera (2010) | Histological score |  | Duodeum, jejunum, ileum, and colon | ileum: 2.36 ± 0.201  colon: 3.31 ± 0.269 | ileum: 0.135 ± 0.219  colon: 0.305 ± 0.243 | at the end of study (day 47) |
| Islam (2017) | Histological score |  | Distal colon | 3.25 ± 0.103 | 2.86 ± 0.291 | After 12 days of DSS induction |
| Kanauchi (2003) | Histological score |  | Proximal, middle, and distal colon | 12.89 ± 3.18* | 6.48 ± 1.68* | 6 days after induction, 13 days after diet |
| Kanauchi (2008) | Histological score |  | Distal colon | 6.76 ± 0.99 | 2.6 ± 0.838 | 7 weeks after cell transfer and 9 weeks after diet |
| Le Leu (2013) | Histological score | Blinded pathologist | Colon sections | 1.01 ± 0.289 | 0.793 ± 0.445 | 7 days after DSS treatment |
| Moreau  (2003) | Histological score using adapted version of Appleyard & Wallace scale | Blinded assessor | Caecum, proximal and distal colon | Caecum: 8.74 ± 0.00  Proximal: 2.27± 0.232  Distal: 4.5964 ± 0.202 | Caecum: 6.40 **±** 0.241  Proximal: 2.04± 0.0231  Distal: 3.71± 0.165 | Day 14 after induction and intervention |
| Moreau (2004) | macroscopic score described by Appleyard and Wallace | Blinded assessor | Ceacal, proximal, distal | day 7: 10.8 ± 0.9  day 14: 8.7 ± 0.6 | day 7: 5.2 ± 1.3  day 14: 5.5 ± 0.8 | At day 7 and day 14 of study |
| Panasevich (2015) | Histological score | Blinded observer | Distal colon | 2.33 ± 0.136 | 1.23 ± 0.209 | at study conclusion (8 days post-DSS) |
| Praengam  (2017) | Visual examination | Two pathologists | Proximal and distal colon | Severity of inflammation decreased significantly in DSS treated mice receiving RS intervention. | | 1 week after disease induction, and 4 weeks after starting treatment |
| Qian  (2013) | Visual examination | One blinded pathologist |  | RS3 + stachyose intervention reduced the lesions of the colon in DSS-induced colitis in the starch | | 1 week after disease induction, and 2 weeks after starting treatment |
| Scarminio (2012) | Histological damage | Blinded observer | Colonic segment | 14.0 ±1.25* | 9.5±1.25* | 1 week after disease induction, and 3 weeks after starting treatment |
| Shende (2020) | Histological damage | Blinded assessor | Proximal and distal colon | Distal: 17.080 ± 0.599  Proximal: 9.463 ± 0.738 | Distal:13.523 ± 0.872  Proximal: 9.262 ± 0.637 | 1 week after disease induction, and 2 weeks after starting treatment |

*Histology scores reported as mean± SD; all other histology scores are reported as mean± SEM.

**Appendix IV – Additional analyses**


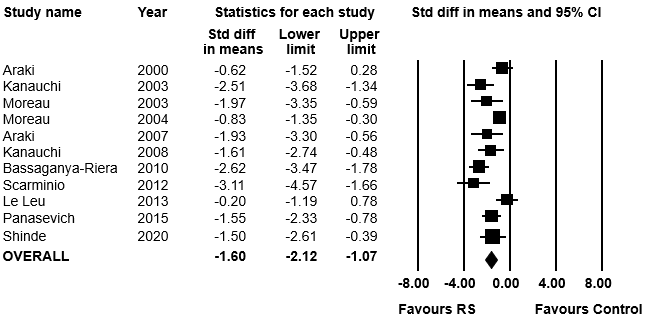


*Supplemental Figure 1. Sensitivity analysis removing extreme values from analysis of standardized mean differences (95%) and pooled estimates for histology score (preclinical studies)*


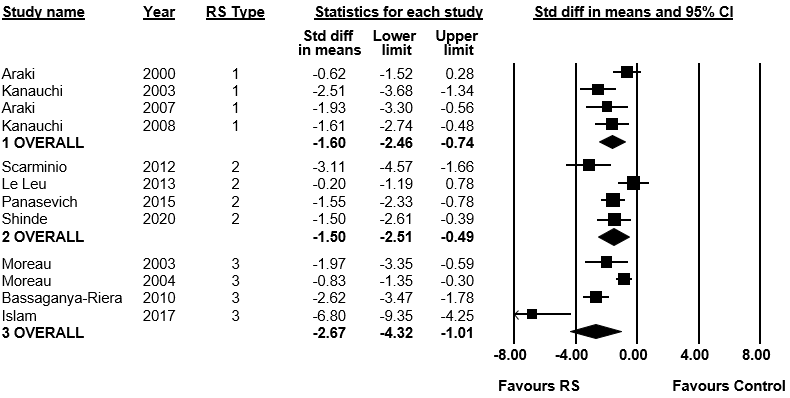


*Supplemental Figure 2. Standardized mean differences (95%) and pooled estimates for histology score (preclinical studies) subgrouped by resistant starch type*


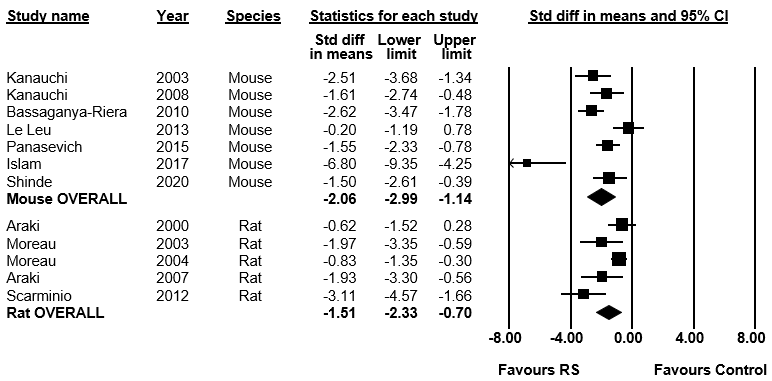


*Supplemental Figure 3. Standardized mean differences (95%) and pooled estimates for histology score (preclinical studies) subgrouped by species*


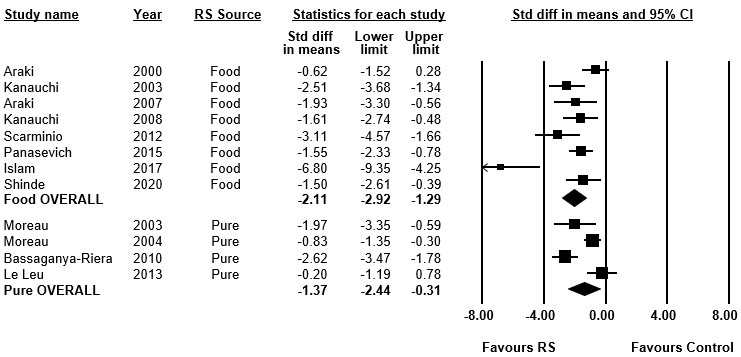


*Supplemental Figure 4. Standardized mean differences (95%) and pooled estimates for histology score (preclinical studies) subgrouped by resistant starch source*

**Appendix V – Publication Bias**


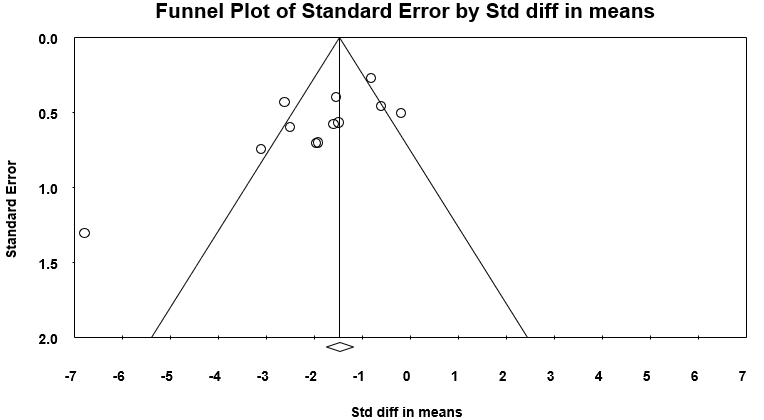


*Supplemental Figure 8. Funnel plot assessing publication bias of studies reporting on histology outcome (n=12). Standardized mean difference (SMD) of histology scores between resistant starch groups compared to placebo or non-active arm is plotted on the x-axis; standard error (SE) of the standardized mean difference is plotted on the y-axis.*

Appendix VI – Risk of Bias


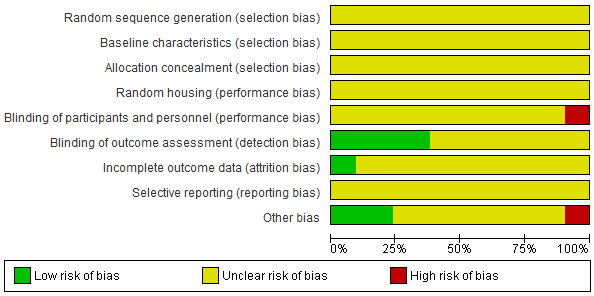


*Supplemental Figure 9. Risk of bias summary for preclinical studies*


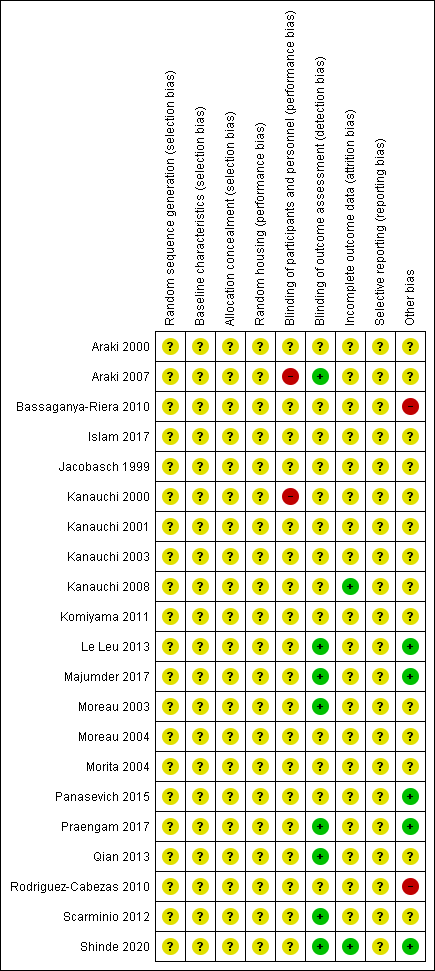


*Supplemental figure 10. Individual study risk of bias assessment for preclinical studies*

*
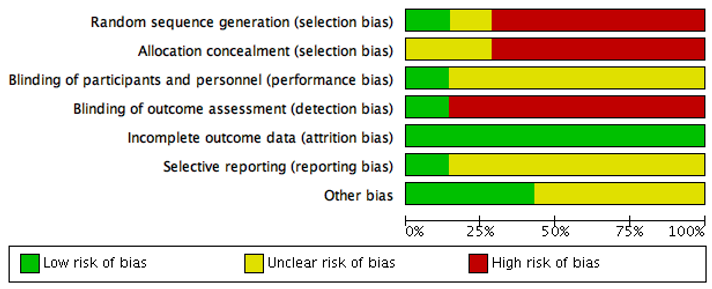
*

*Supplemental figure 11. Risk of bias summary for clinical studies*


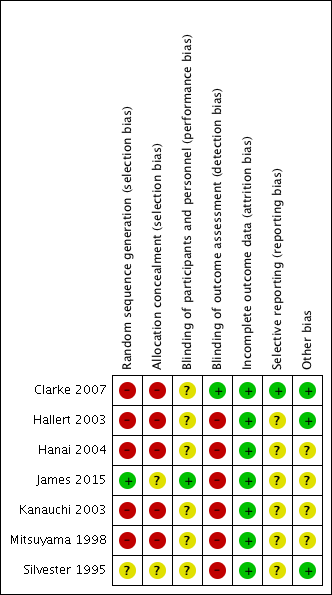


*Supplemental figure 12. Individual study risk of bias assessment for clinical studies*
